# Effects of exercise dose based on the ACSM recommendations on pain and disability in non-specific low back pain patients: a systematic review and meta-analysis of randomized controlled trials

**DOI:** 10.3389/fphys.2026.1725132

**Published:** 2026-03-11

**Authors:** Tao Tan, Xiang Tan, Jiyong Xie, Bo Zeng, Jincen Xie, Huan Wang, Fei Wen

**Affiliations:** Department of Orthopedics, The People’s Hospital of Rongchang District, Chongqing, China

**Keywords:** ACSM, exercise, meta-analysis, non-specific low back pain, systematic review

## Abstract

**Background:**

Low back pain (LBP) is characterized by pain or discomfort between the costal margins and the inferior gluteal folds, with or without radiation to the lower limbs. It significantly affects patients’ overall health and quality of life.

**Purpose:**

This study aims to investigate the effects of exercise therapy and adherence to the American College of Sports Medicine (ACSM) guidelines on treatment outcomes in patients with LBP.

**Methods:**

The literature search, concluded on 26 June 2025, included studies that investigated the effects of exercise interventions in patients diagnosed with LBP and that provided sufficient data for calculating the Standardized Mean Difference (SMD). The primary outcome measure was the Visual Analogue Scale (VAS), and secondary outcomes included the Oswestry Disability Index (ODI) and the Roland-Morris Disability Questionnaire (RDQ/RMDQ).

**Results:**

Among 22,723 records, 36 studies (n = 2,284) were eligible for qualitative synthesis. The meta-analysis showed an overall SMD for pain of −1.28 (95% CI -1.63, −0.94). In the subgroup with high adherence to the ACSM guidelines, the pooled SMD was −1.98 (95% CI -2.58, −1.38), whereas in the low- or uncertain-adherence subgroup it was −0.72 (95% CI -1.01, −0.43). For disability, the overall SMD was −1.10 (95% CI -1.58, −0.62) on the ODI. High adherence yielded a mean difference of −1.31 (95% CI -1.81, −0.80) and low adherence yielded −0.99 (95% CI -1.71, −0.28). The overall SMD was −0.77 (95% CI -1.07, −0.47) on the RDQ; the corresponding values were −1.39 (95% CI -2.51, −0.26) for high adherence and −0.54 (95% CI -0.76, −0.31) for low adherence.

**Conclusion:**

The results suggest that exercise interventions with high adherence to the ACSM recommendations are more effective in improving pain and disability in individuals with LBP than interventions with low or uncertain adherence to these guidelines.

## 1 Introduction

LBP is characterized by pain or discomfort between the costal margins and the inferior gluteal folds, with or without radiating pain in the lower limbs. It is strongly associated with disability, work absenteeism, and mood disorders such as depression and anxiety (Escorpizo, 2014; Mák et al., 2021). In the United Kingdom, approximately £12 billion is spent annually on direct medical and non-medical costs, as well as indirect costs related to productivity loss and absenteeism (Dagenais et al., 2008). A recent inception cohort study demonstrated that 40% of patients with acute LBP seen in primary care settings progress to chronic LBP, highlights both the high prevalence of the condition and its substantial impact on patients’ quality of life and socioeconomic status, underscoring the urgent need for more effective treatment strategies (El-Haddad et al., 2018). Current treatment approaches include pharmacological therapies - such as non-steroidal anti-inflammatory drugs (e.g., ibuprofen, celecoxib) for pain and inflammation, muscle relaxants (e.g., eperisone) for spasms, and neurotropic agents (e.g., mecobalamin) for nerve root compression - and non-pharmacological interventions like exercise, smoking cessation, and reduced alcohol consumption (El-Haddad et al., 2018; Jiang et al., 2022; Golovacheva et al., 2023). However, pharmacological treatments often carry side effects that may indirectly reduce quality of life. Exercise, as a key non-pharmacological intervention, not only alleviates LBP symptoms but also enhances overall well-being and quality of life (Suh et al., 2019).

Research has shown that following an exercise intervention, individuals with LBP experience significant improvements in quality of life, pain scores, and balance performance (Dal Farra et al., 2022). Exercise therapy is recommended by clinical practice guidelines as an effective intervention for the treatment of non-specific LBP. Pain and function are critical diagnostic indicators in individuals with LBP, and exercise plays a key role in reducing pain and improving function for both prevention and treatment (Hayden et al., 2005). The most recent Cochrane systematic review on exercise for chronic low back pain (CLBP) concluded that all forms of exercise therapy are equally effective and at least as efficacious as other conservative treatments (Hayden et al., 2005). Exercise includes a wide range of modalities, such as aerobic, resistance, strength, balance, and proprioceptive training. Consequently, the studies exhibited significant heterogeneity due to the wide variety of exercise protocols used. Moreover, considerable variation in the number of control groups incorporated into the studies due to the limited availability of specific control therapies, potentially compromising the overall quality of evidence. In contrast, the ACSM provides comprehensive, systematic, and standardized exercise guidelines with predefined minimum dose thresholds for aerobic, resistance, and flexibility exercises. These guidelines specify evidence-based recommendations for exercise frequency, intensity, time, and type (FITT principles), including minimum intensity thresholds: moderate-intensity aerobic exercise (40%–60% heart rate reserve or 64%–76% maximum heart rate), resistance training involving 1 sets of 8–12 repetitions at 60%–70% one-repetition maximum, and flexibility exercises held for 10–30 s per stretch. Specifically for chronic disease populations including those with musculoskeletal conditions, the ACSM guidelines emphasize individualized progression while maintaining these minimum dosage thresholds to ensure physiological adaptation and therapeutic benefit. A recent randomized controlled trial found that high-intensity aerobic training was more effective than moderate-intensity training in reducing pain and improving function in individuals with LBP (Verbrugghe et al., 2021). Although exercise is an effective non-pharmacological treatment for LBP, the optimal exercise dosage for both prevention and treatment remains unclear, and further experimental evidence is needed to establish definitive recommendations.

The effectiveness of exercise for LBP is thought to depend not only on dosage parameters but also on proper exercise form and core activation. Proper form ensures that targeted muscle groups are effectively engaged while minimizing compensatory movements that may exacerbate pain or injury risk. Core activation, involving the coordinated contraction of deep stabilizing muscles including the transversus abdominis, multifidus, and pelvic floor muscles, is considered fundamental to spinal stability and load distribution during movement. Emerging evidence suggests that exercises emphasizing core activation may provide superior outcomes for LBP by enhancing neuromuscular control and reducing abnormal spinal loading. However, the extent to which the exercise programs reviewed in existing literature explicitly addressed form supervision and core activation remains unclear, as these details are often inadequately reported in primary studies. This represents an important gap in the current evidence base, as the therapeutic potential of exercise may be substantially influenced by the quality of movement execution rather than dosage alone.

Despite the established benefits of exercise for LBP, significant gaps remain in understanding how adherence to standardized exercise guidelines influences treatment outcomes. Previous systematic reviews have primarily focused on comparing different exercise modalities without adequately addressing the critical issue of guideline adherence (Zang and Yan, 2024). The heterogeneity in exercise prescriptions across studies - characterized by variations in frequency, intensity, time, and type (FITT principles) - has limited the ability to draw definitive conclusions regarding optimal exercise parameters for LBP management. Furthermore, while the ACSM guidelines provide evidence-based recommendations for exercise prescription in healthy populations and various clinical conditions, their specific application to LBP populations requires further investigation. Recent evidence suggests that structured exercise programs adhering to established guidelines may yield superior outcomes compared to unstructured or poorly defined interventions; however, this hypothesis has not been systematically evaluated in the LBP literature (Zang and Yan, 2024). Therefore, this study aims to comprehensively evaluate how adherence to ACSM exercise guidelines influences treatment outcomes in individuals with LBP. By systematically examining the relationship between guideline-concordant exercise prescription and clinical outcomes, we seek to establish a foundation for more precise and personalized exercise prescription in clinical practice. Ultimately, this approach may enhance the translational value of exercise interventions and optimize patient-centered care for individuals suffering from low back pain.

## 2 Materials and methods

The systematic review and meta-analysis will be conducted in accordance with the Preferred Reporting Items for Systematic Reviews and Meta-Analyses (PRISMA) statement and registered with PROSPERO (CRD420251139872).

### 2.1 Search strategy

We searched PubMed, Embase, Web of Science, and Cochrane databases from inception to 26 June 2025, using a search strategy based on the PICOS framework, focusing on the study population, intervention, and research methodology. Both Medical Subject Headings (MeSH) and free-text terms for “Exercise,” “Low back pain,” and “Randomized controlled trial” were combined with appropriate Boolean operators.

The detailed search strategy is presented in Supplementary Material 1. We also manually screened the reference lists of relevant reviews and included studies. When required, we contacted study authors for additional information.

### 2.2 Criteria for selection of studies

We included studies that met the following criteria: (a) published randomized controlled trials (RCTs); (b) study participants were individuals with LBP; (c) the intervention could be any land-based exercise program, including resistance training, aerobic exercise, flexibility exercise, etc.,; (d) the control intervention could be no treatment or any treatment unrelated to exercise; thus, studies comparing different exercise interventions were excluded; (e) reporting of the VAS, ODI, or RDQ in the study results.

We excluded the following studies: (a) reports, conference proceedings, magazines, reviews, etc.,; (b) studies based on aquatic exercise and those without a comparison between a land-based exercise intervention and a non-exercise group; (c) populations with confirmed radiculopathy due to herniated discs, acute spinal fractures, tumors, pregnancy, or signs of progressive neurological deficits; (d) studies that involved special drug treatments during the exercise intervention; (e) duplicate experimental data from multiple publications of the same study.

Two authors (TT and BZ) will independently screened the titles and abstracts of the literature that met the inclusion criteria. If one of the authors considered a study to meet the criteria, the full text of the article will be obtained. Then, two authors independently assessed whether the full text met the requirements. In case of disagreement, the third author (HW) made a final decision, and consensus was reached through discussion. There were no restrictions on participant age, gender, body mass index, publication date, or language.

### 2.3 Data synthesis and analysis

Data were extracted independently by two reviewers (TT and BZ) using a pre-designed Excel spreadsheet. The primary outcome was lumbar pain intensity measured with the VAS; secondary outcomes were functional disability assessed using the ODI and RDQ. Extracted variables comprised study identifiers (title, authors, country, year), design elements (number of groups, group descriptions, intervention details, sample size), participant characteristics (age, sex distribution, body mass index [BMI]), exercise parameters (frequency, intensity, duration, repetitions, sets), risk-of-bias items, and outcome data.

When extracting outcome data, if the study did not clearly report post-intervention results but presented them in graphical form, Engauge Digitizer software was used to extract the data. For studies with multiple follow-up evaluations, we extracted only the data immediately after the intervention.

After data were extracted, we evaluated the dose and adherence of each exercise intervention. The dose was assessed against the ACSM recommendations for developing and maintaining cardiorespiratory, musculoskeletal, and neural function in individuals with LBP (Garber et al., 2011). Two authors (TT and BZ) independently assessed each study’s exercise intervention against ACSM criteria for frequency, intensity, duration, and other relevant aspects to evaluate adherence to exercise dose (Table 1).

**TABLE 1:** The American College of Sports Medicine (ACSM) recommendations for cardiorespiratory fitness, muscular strength and flexibility in apparently healthy adults.

Exercise dose	Cardiorespiratory exercise	Resistance exercise	Flexibility exercise
Frequency	≥3–5 days/week	1–2 days/week (non-consecutive days), gradually increasing to 2–3 days/week	5–7days/week
Intensity/workload	40%–60% VO2R or HRR; 64%–76% HRmax; RPE of 12–13 on a 6–20 scale	Adjust resistance, medium to high intensity (Start with 40%–50% 1RM, more capable with 60%–70% 1RM)	Stretch until you feel your muscles being pulled tight or a slight discomfort
Duration	Gradually increase from 20 min to at least 30 min (up to 45–60 min)	Starting with one set of 8–12 repetitions, increase to two sets after about 2 weeks. Perform no more than 8–10 exercises per session	Static stretching held for 10–30 s, repeated 2–4 times

HRR, Heart rate reserve. VO^2^R, Oxygen uptake reserve. 1RM, one repetition maximum.

The scoring range for each exercise indicator was 0–2 points. 2 points were assigned for fully meeting the criteria, 1 point for partial or ambiguous adherence, and 0 points for not meeting the criteria. In cases of disagreement during the evaluation process, the researchers consulted a third author to reach consensus. Using this scoring method, the proportion of exercise dose adherence conforming to ACSM guidelines was calculated for each study as follows: the sum of actual scores for all exercise indicators was divided by the maximum possible score (number of exercise indicators *2) and multiplied by 100%. A proportion rate of ≥75% was considered strong adherence to ACSM recommendations, while <75% indicated low or questionable adherence (Cui et al., 2023; Han et al., 2024; Luan et al., 2024).

### 2.4 Statistical analyses

Meta-analysis was performed using Review Manager 5.4.1 and Stata 12.0. For non-normally distributed data reported as medians (M) with interquartile ranges (P25, P75), we converted these to mean ± standard deviation (SD) using the method described by McGrath (McGrath et al., 2020). The SMD served as the effect measure, and heterogeneity was quantified with the Higgins I^2^ statistic. Because exercise modality, frequency, intervention length, and single-session duration varied across trials, we *a priori* specified a random-effects model. When I^2^ indicated considerable heterogeneity, meta-regression was undertaken to identify potential sources. Publication bias was appraised with funnel plots, Begg’s rank-correlation test, and Egger’s linear regression (p < 0.05 considered significant). In the presence of publication bias, we used the trim-and-fill method to assess its impact on the meta-analysis results. This method involves iteratively estimating the number of missing or unpublished studies and recalculating the effect size to determine whether the overall findings remain robust. Sensitivity analyses were also performed to examine the robustness of the study results by excluding each study one by one.

### 2.5 Quality appraisal

The methodological quality of the included studies was assessed by two pairs of reviewers (TT and BZ, JCX and HW) using the quality assessment criteria recommended by the Cochrane Collaboration (Higgins et al., 2011). The recommended tool is the original Cochrane Risk of Bias (RoB 1) tool (Zang et al., 2024). The RoB 1 tool provides a framework for assessing the risk of bias for individual outcomes in any type of randomized trial. The evaluation indicators include random sequence generation (addressing selection bias), allocation concealment (addressing performance bias), blinding of participants and personnel (addressing performance bias), blinding of outcome assessment (addressing detection bias), incomplete outcome data (addressing attrition bias), selective reporting (addressing reporting bias), and other biases. Reviewers rate the studies based on the Cochrane Handbook. The bias risk for each area is categorized into three levels: “low risk,” “some concerns,” and “high risk.” If all areas are evaluated as “low risk,” the overall bias risk is considered “low.” If some areas are evaluated as “some concerns” and no areas are rated as “high risk,” the overall bias risk is considered “some concerns.” If any area is rated as “high risk,” the overall bias risk is considered “high.” (Cumpston et al., 2019).

## 3 Results

### 3.1 Study selection

A total of 22,723 records were retrieved from four databases: PubMed (2,317), Cochrane Library (5,419), Embase (2,387), and Web of Science (12,596), with an additional four records manually identified from other sources. After removing 11,262 duplicates, 11,461 articles remained.

**FIGURE 1:**
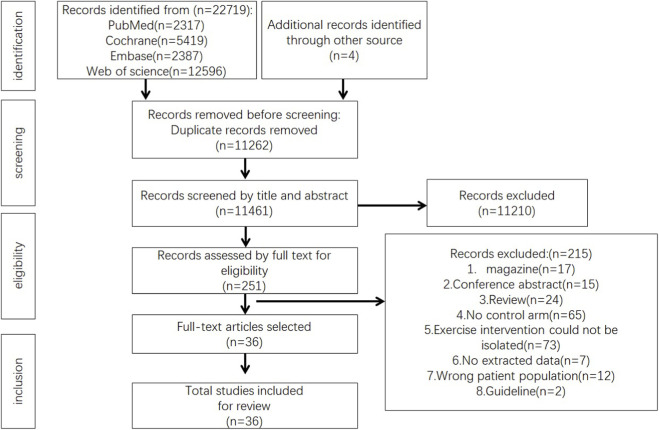
PRISMA study flow diagram.

Following title and abstract screening, 251 articles were selected for full-text review, and ultimately, 36 articles were included in this review (Figure 1). These studies are as follows: (Dettori et al., 1995; Shaughnessy and Caulfield, 2004; Williams et al., 2005; Gladwell et al., 2006; Harts et al., 2008; Williams et al., 2009; Hall et al., 2011; Tilbrook et al., 2011; Bruce-Low et al., 2012; Miyamoto et al., 2012; Steele et al., 2013; Cho et al., 2014; Natour et al., 2014; Zhang et al., 2015; Tang et al., 2016; Valenza et al., 2016; Cortell-Tormo et al., 2017; Groessl et al., 2017; Lopes et al., 2017; Bellido-Fernández et al., 2018; Cruz-Díaz et al., 2018; Kuvačić et al., 2018; Noormohammadpour et al., 2018; Liu et al., 2019; Prado et al., 2019; Abass et al., 2020; Narouei et al., 2020; Bellido-Fernández et al., 2021; Hatefi et al., 2021; Ye et al., 2021; Arya et al., 2022; Ge et al., 2022; Lin et al., 2022; Jahandideh et al., 2023; Pinho et al., 2023; Kosić et al., 2024) (Table 2).

**TABLE 2:** Basic characteristics of the included studies.

Author, year	Country	Age (years)		Sample size (n)		BMI (Kg/m2)		Interventions	Length of intervention
		IG	CG	IG (F:M)	CG (F:M)	IG	CG		
Abass et al. (2020)	Nigeria	48.15 (9.02)	53.1 (7.91)	20 (14:6)	20 (15:5)	29.51 (15.99)	26.57 (5)	Stabilization exercise	8 weeks
Hall et al. (2011)	Australia	43.4 (13.5)	44.3 (13)	80 (63:17)	80 (56:24)	NR	NR	Tai Chi exercise	10 weeks
Cruz-Díaz et al. (2018)	Spain	37.9 (8.2)	35.6 (6.7)	32 (21:11)	30 (20:10)	22.38 (2.71)	20.77 (2.57)	Pilates	12 weeks
Prado et al. (2019)	Brazil	35 (9.8)	33 (11.3)	27 (19:8)	27 (17:10)	25.17 (3.6)	25.42 (3.62)	Isostretching	45 days
Miyamoto et al. (2012)	Brazil	40.7 (11.8)	38.3 (11.4)	43 (36:7)	43 (34:9)	25.5 (4.9)	24.6 (4.0)	Modified pilates	6 weeks
Pinho et al. (2023)	Portugal	38.8 (12)	45.1 (13.6)	25 (12:13)	24 (15:9)	NR	NR	Aerobic exercise	NR
Cho et al. (2014)	Korea	38.1 (7.9)	36.5 (7.7)	15 (9:6)	15 (10:5)	NR	NR	CORE exercise	4 weeks
Natour et al. (2014)	Brazil	47.79 (11.47)	48.08 (12.98)	30 (24:6)	30 (23:7)	23.1 (4.9)	24.3 (4.5)	Pilates	90 days
Liu et al. (2019)	China	58.13 (5.38)	60.67 (2.58)	15 (11:4)	13 (10:3)	NR	NR	Chen-Style Tai Chi	12 weeks
Cortell-Tormo et al. (2017)	Spain	35.6 (7.9)	35.6 (9.7)	11 (11:0)	8 (8:0)	23.8 (2.3)	24.3 (2.4)	Resistance training	12 weeks
Williams et al. (2005)	USA	48.7 (10.6)	48 (1.96)	20 (13:7)	24 (17:7)	NR	NR	Lyengar yoga	16 weeks
Williams et al. (2009)	USA	48.4 (1.86)	47.6 (1.47)	43 (32:11)	47 (37:10)	25.8 (0.57)	27.4 (0.60)	Lyengar yoga	24 weeks
Ge et al. (2022)	China	64.60 (3.71)	64.12 (2.96)	15 (15:0)	16 (16:0)	23.34 (1.37)	22.72 (1.20)	Core stability training	4 weeks
Ye et al. (2021)	Korea, China	42.63 (9.60)	41.45 (10.23)	42 (19:23)	42 (18:24)	NR	NR	Suspension training	1 month
Jahandideh et al. (2023)	Iran	36.92 (5.39)	37.13 (3.61)	15 (11:4)	15 (9:6)	26.12 (3.04)	25.53 (2.32)	Gluteal-to-tensor fasciae latae activation	8 weeks
Valenza et al. (2016)	Spain	26.39 (11.57)	28.17 (8.63)	27 (5:22)	27 (2:25)	26.39 (11.57)	28.17 (8.63)	Pilates	8 weeks
Hatefi et al. (2021)	Iran	26.27 (2.13)	26.43 (2.57)	15 (15:0)	15 (15:0)	23.15 (3.15)	23.32 (4.25)	Static stretching exercises	8 weeks
Noormohammadpour et al. (2018)	Iran	43.3 (7.5)	41.3 (6.4)	10 (10:0)	10 (10:0)	24 (1.7)	24.3 (2.1)	Core stability exercises	8 weeks
Narouei et al. (2020)	Iran	32.23 (6.32)	32.13 (6.96)	17 (12:5)	15 (9:6)	24.72 (4.59)	26.47 (4.38)	Core stabilization exercises	4 weeks
Tang et al. (2016)	China	41.7 (5.6)	43.6 (6.4)	41 (10:31)	41 (13:28)	NR	NR	Core stability exercises	6 weeks
Bruce-Low et al. (2012)	UK	45.5 (14.1)	45.5 (14.1)	20(NR)	21(NR)	NR	NR	Lumbar extension training	12 weeks
Lopes et al. (2017)	Portugal	21.8 (3.2)	22.8 (3.6)	23 (13:10)	23 (14:9)	22.1 (2.4)	22.2 (3.2)	Pilates	NR
Arya et al. (2022)	India	42.5 (12.6)	42.5 (12.7)	45(NR)	40(NR)	30.44 (4.97)	30.65 (2.9)	Medical yoga therapy	8 weeks
Gladwell et al. (2006)	UK	36.9 (8.1)	45.9 (8.0)	20 (17:3)	14 (10:4)	NR	NR	Pilates	6 weeks
Zhang et al. (2015)	China	48.71 (3.89)	51.62 (4.03)	43(NR)	42(NR)	NR	NR	Core stability exercises	8 weeks
Shaughnessy and Caulfield (2004)	Ireland	43 (9)	46 (11)	20 (14:6)	21 (13:8)	NR	NR	Stabilisation exercise training	10 weeks
Lin et al. (2022)	Taiwan	45.5 (10.74)	41.95 (11.85)	24 (22:2)	22 (21:1)	23.55 (2.92)	24.42 (4.68)	Core stability exercise	8 weeks
Kuvačić et al. (2018)	Croatia	33.6 (4.30)	34.7 (4.83)	15 (6:9)	15 (8:7)	NR	NR	Yoga	8 weeks
Bellido-Fernández et al. (2018)	Spain	36.67 (25.93)	26 (8.15)	9 (8:1)	9 (6:3)	NR	NR	Abdominal hypopressive gymnastics	5 weeks
Bellido-Fernández et al. (2021)	Spain	34 (20.74)	43 (23.7)	20 (14:6)	20 (15:5)	23.8 (2.4)	25.7 (4)	Abdominal hypopressive gymnastics	5 weeks
Groessl et al. (2017)	California	53.3 (12.7)	53.6 (13.9)	75 (20:55)	75 (19:56)	NR	NR	Yoga	12 weeks
Tilbrook et al. (2011)	UK	46.4 (11.3)	46.3 (11.5)	156 (106:50)	157 (114:43)	NR	NR	Yoga	12 weeks
Kosić et al. (2024)	Croatia	58.3 (15.4)	58.3 (15.5)	30(NR)	30(NR)	22.6 (3.06)	22.6 (3.07)	Lumbosacral kinesiotherapy	10 days
Dettori et al. (1995)	Germany	29.6	26.1	119 (96:23)	30 (24:6)	NR	NR	Flexion and extension exercises	1 Week
Steele et al. (2013)	UK	46 (12.36)	41.7 (15.1)	10(NR)	7(NR)	25.2 (3.15)	25.94 (4.41)	Motion lumbar extension exercise	12 weeks
Harts et al. (2008)	Netherlands	44 (10)	41 (9)	23 (23:0)	21 (21:0)	NR	NR	Extension exercises	8 weeks

IG, intervention group; CG, control group; Numbers are mean (SD) unless otherwise stated; NR, not reported; F, female; M, male.

### 3.2 Study characteristics

The 36 articles each reported one comparative study, collectively enrolling 2,284 participants (1,195 in the intervention and 1,089 in the control groups). Geographically, the trials were conducted in: China and Spain (five each), Iran and the United Kingdom (four each), Brazil (three), the United States (three, including one in California), Portugal, Croatia and South Korea (two each), and Taiwan, Nigeria, Ireland, India, Australia, Germany and the Netherlands (one each). The article by Ye et al. covered sites in both China and South Korea (Ye et al., 2021). Intervention duration ranged from 10 days to 12 weeks, and session frequency ranged from 2 to 7 days per week. All programmes were either supervised or home-based, and comprised resistance exercise, balance training, yoga, Tai Chi, stabilization exercises, Pilates or aerobic exercise. Regarding exercise dosage based on ACSM recommendations, 35 studies addressed aerobic exercise, 2 studies focused on resistance exercise, and 33 studies involved flexibility exercises. Full details are given in Table 2.

### 3.3 Risk of bias

Among the 36 studies included, 28 demonstrated a low risk of bias in random sequence generation. Six studies were rated as having an unclear risk due to insufficient description of the randomization process, while two were classified as high risk because of non-random allocation. Regarding allocation concealment, 15 studies were deemed low risk, 14 had an unclear risk due to inadequate reporting, and 7 were considered high risk.

Blinding presented significant challenges: the inherent difficulty in implementing double-blinding for exercise interventions resulted in elevated risks for both researchers and participants. Twenty-five studies employed blinded outcome assessors, indicating a low risk; five lacked clarity in assessment methods, raising concerns; and six studies with non-blinded assessors were categorized as high-risk.

**FIGURE 2:**
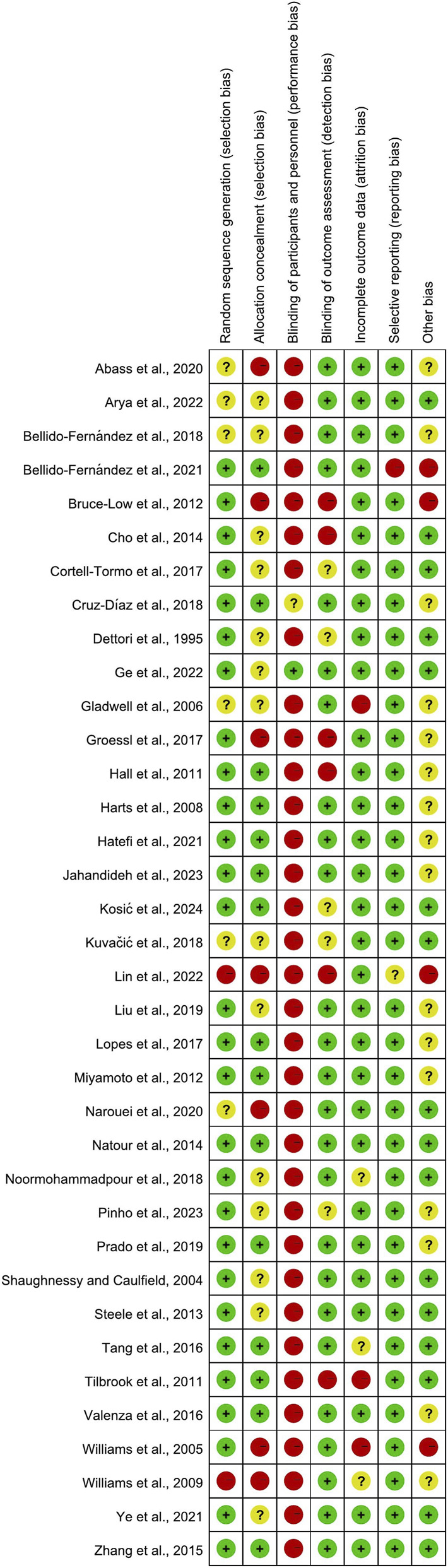
Risk of bias summaries for all exercise trials.

**FIGURE 3:**
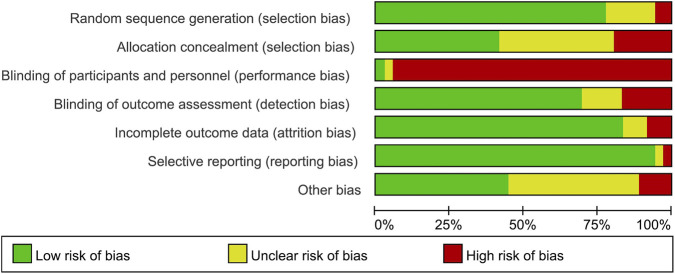
Combined percentage risk of bias in each risk domain for all included trials.

Incomplete outcome data affected six studies, with three posing a moderate risk (minor participant withdrawals) and three exhibiting a high risk (substantial inter-group attrition). Selective reporting bias was low in 34 studies. One study had an unclear risk (unregistered protocol or inadequate withdrawal documentation), and one was considered high-risk (absence of predefined analysis plans). Additionally, 20 studies exhibited “other bias” sources: 16 with unclear risk and 4 with high risk (Figures 2, 3).

### 3.4 Compliance with the ACSM recommendations

We employed a standardized scoring system to evaluate compliance with the ACSM guidelines. Intervention groups were classified as high-compliance (≥75% adherence) or low-compliance based on exercise prescription. Among the 36 trials, 15 demonstrated high compliance (Dettori et al., 1995; Williams et al., 2005; Williams et al., 2009; Cho et al., 2014; Cortell-Tormo et al., 2017; Cruz-Díaz et al., 2018; Kuvačić et al., 2018; Liu et al., 2019; Narouei et al., 2020; Hatefi et al., 2021; Ye et al., 2021; Arya et al., 2022; Ge et al., 2022; Jahandideh et al., 2023; Kosić et al., 2024). The remaining 21 studies did not meet this threshold (Shaughnessy and Caulfield, 2004; Gladwell et al., 2006; Harts et al., 2008; Hall et al., 2011; Tilbrook et al., 2011; Bruce-Low et al., 2012; Miyamoto et al., 2012; Steele et al., 2013; Natour et al., 2014; Zhang et al., 2015; Tang et al., 2016; Valenza et al., 2016; Groessl et al., 2017; Lopes et al., 2017; Bellido-Fernández et al., 2018; Noormohammadpour et al., 2018; Prado et al., 2019; Abass et al., 2020; Bellido-Fernández et al., 2021; Lin et al., 2022; Pinho et al., 2023) (see Table 3). Low compliance was attributable to two main deficiencies: either key components of the ACSM prescription were omitted, or the published exercise details were insufficient for full assessment. When stratified by outcome, 26 trials used the VAS (12 high, 14 low compliance), 17 used the ODI or similar disability scales (7 high, 10 low), and 14 used the RDQ (4 high, 10 low); numbers exceed 36 because several studies reported multiple outcomes.

**TABLE 3:** Assessment of ACSM compliance.

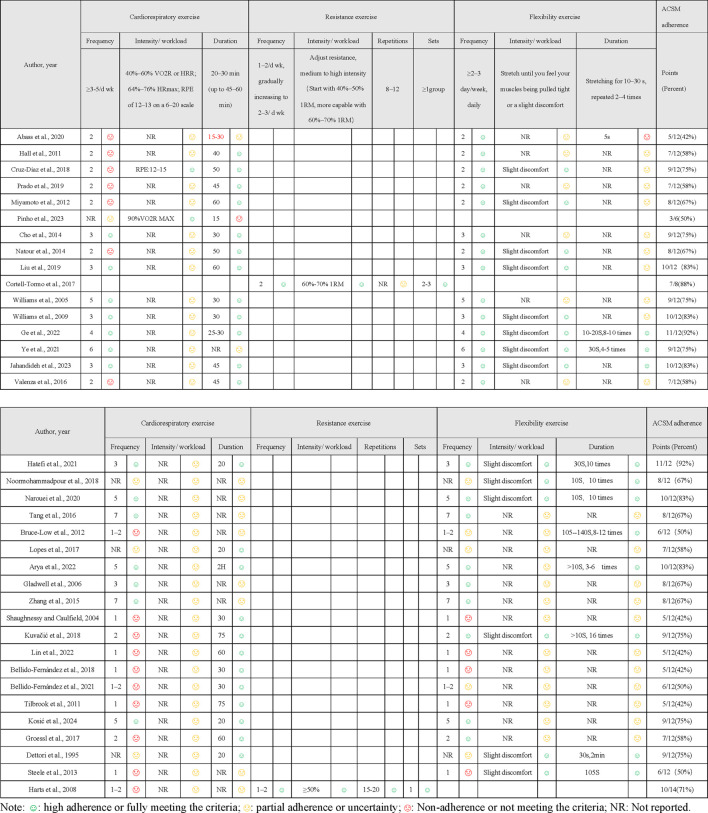

### 3.5 Meta-analysis

**FIGURE 4:**
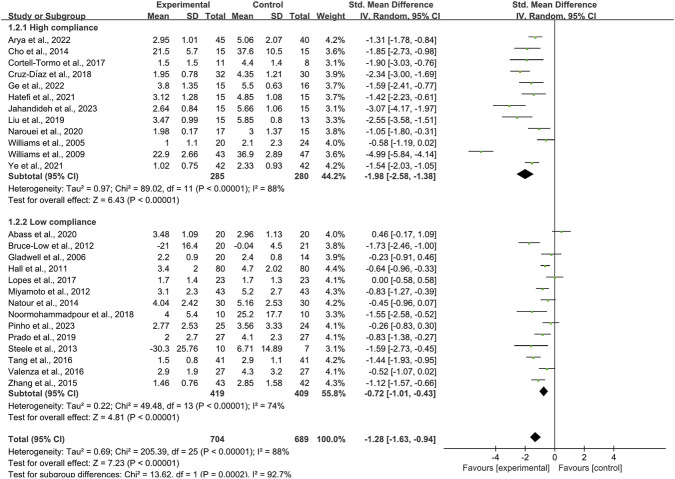
Forest plot of meta-analysis on the effect of exercise on VAS in LBP patients.

**FIGURE 5:**
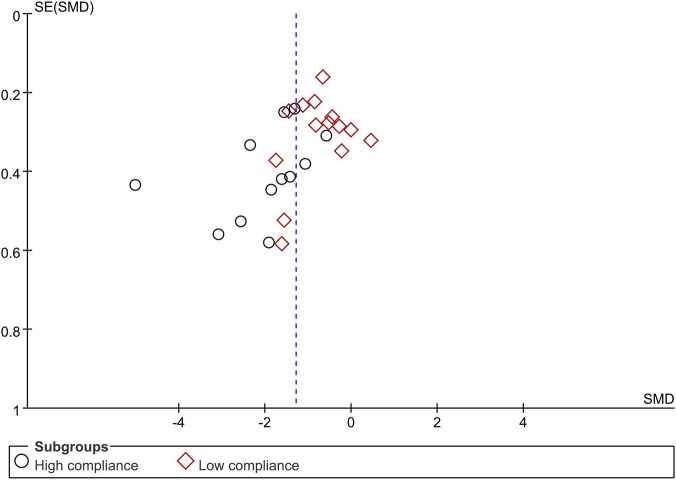
Funnel plot of meta-analysis on the effect of exercise on VAS in LBP patients.

**FIGURE 6:**
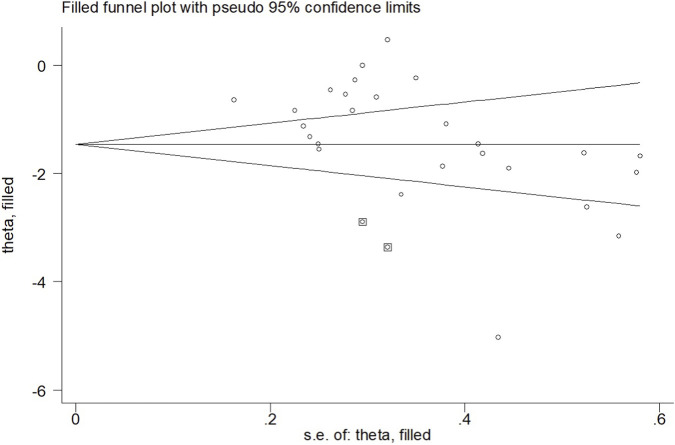
Trim and fill (VAS).

**FIGURE 7:**
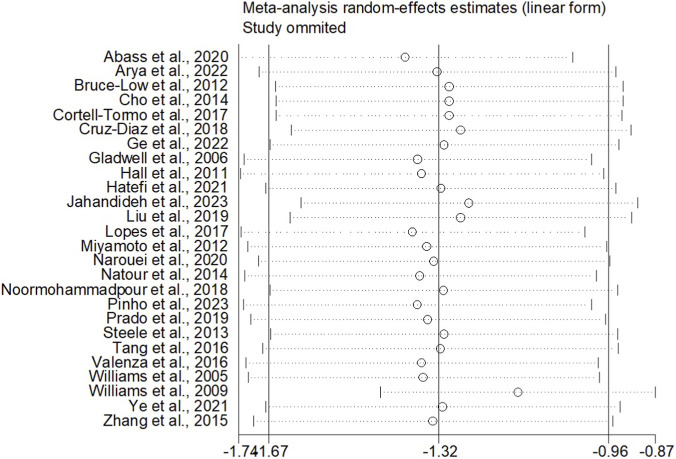
Sensitivity analyses (VAS).

When the outcome was assessed with the VAS, 26 trials enrolling 1,657 participants were included. Twelve trials achieved high adherence to ACSM recommendations, whereas 14 exhibited low or indeterminate adherence. The overall pooled SMD for pain reduction was −1.28 (95% CI: −1.63, −0.94), confirming a statistically significant benefit of exercise. Subgroup analyses revealed a larger effect in the high-adherence stratum (SMD −1.98; 95% CI: −2.58, −1.38) than in the low/indeterminate-adherence stratum (SMD −0.72; 95% CI: −1.01, −0.43); the between-group difference was statistically significant (p < 0.05) (Figure 4). Visual inspection of the funnel plot indicated symmetry (Figure 5). Nevertheless, both Begg’s (P = 0.029) and Egger’s (P = 0.014) tests suggested publication bias. Trim-and-fill analysis imputed two putative missing studies and produced a symmetric plot (Figure 6). Sensitivity analysis demonstrated that no single study materially altered the pooled estimate (Figure 7), corroborating the robustness of the findings.

**FIGURE 8:**
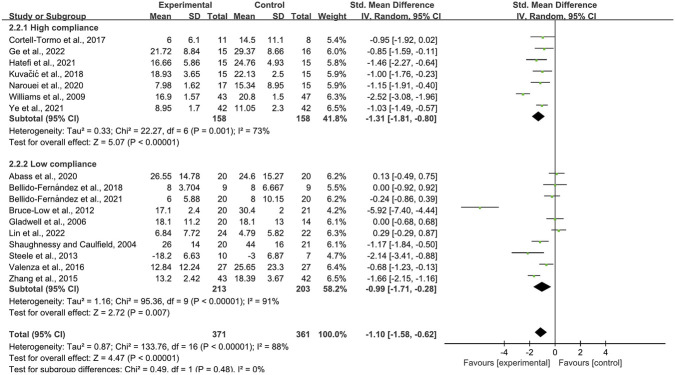
Forest plot of meta-analysis on the effect of exercise on ODI in LBP patients.

**FIGURE 9:**
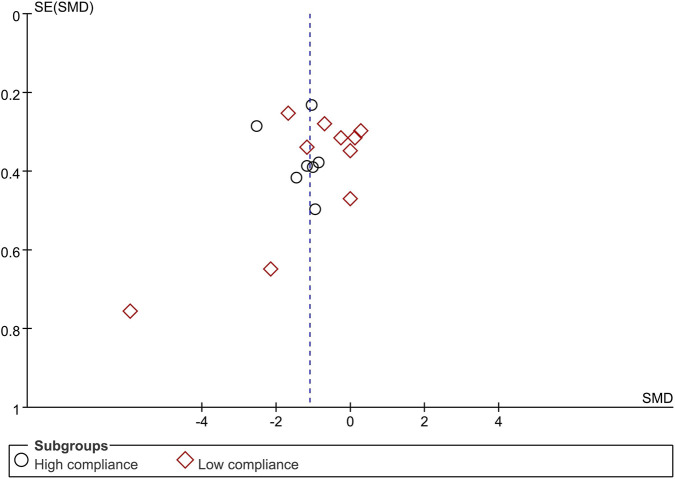
Funnel plot of meta-analysis on the effect of exercise on ODI in LBP patients.

**FIGURE 10:**
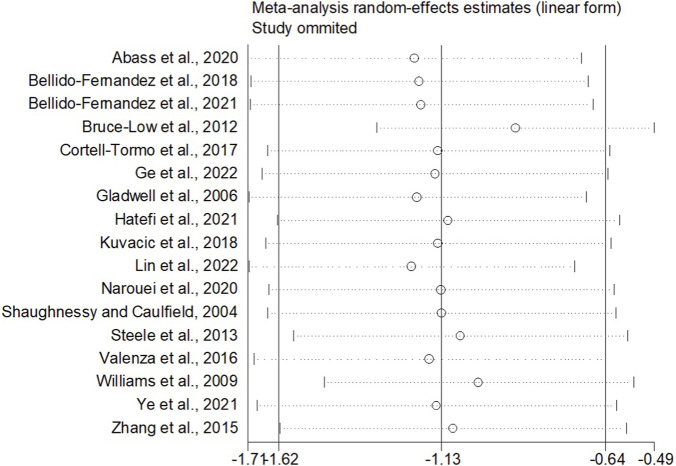
Sensitivity analyses (ODI).

When disability was measured with the ODI, 17 trials enrolling 735 participants were included. Seven trials achieved high adherence to ACSM recommendations; ten exhibited low or indeterminate adherence. The pooled SMD was −1.10 (95% CI: −1.58, −0.62), indicating a statistically significant improvement. In the high-adherence stratum the SMD was −1.31 (95% CI: −1.81, −0.80), whereas in the low/indeterminate-adherence stratum it was −0.99 (95% CI: −1.71, −0.28). The between-group difference was statistically significant (p < 0.05) (Figure 8). The funnel plot appeared symmetric (Figure 9), and neither Begg’s (P = 0.249) nor Egger’s (P = 0.304) test suggested publication bias. Sensitivity analysis confirmed that no single study materially influenced the pooled estimate (Figure 10).

**FIGURE 11:**
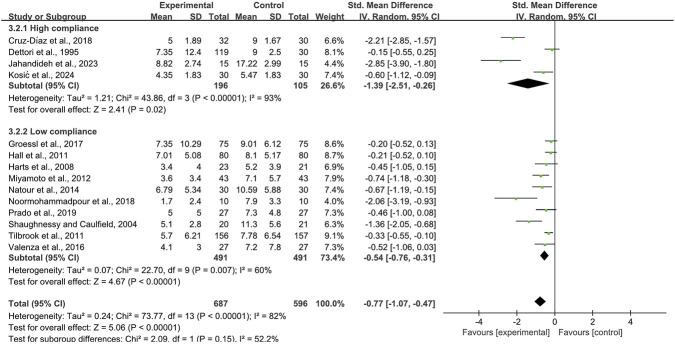
Forest plot of meta-analysis on the effect of exercise on RDQ in LBP patients.

**FIGURE 12:**
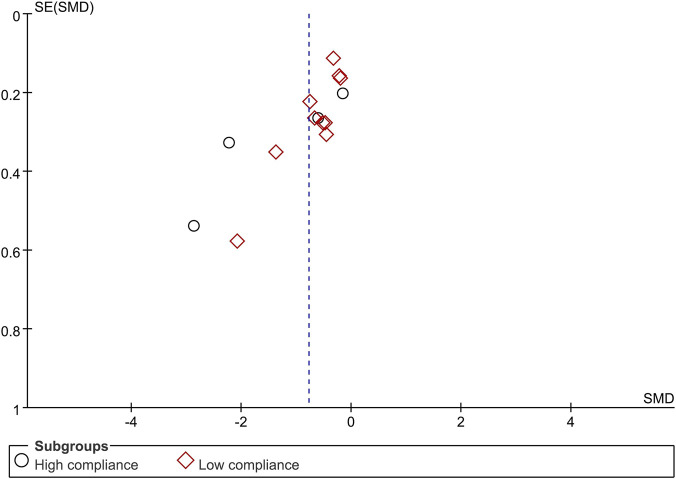
Funnel plot of meta-analysis on the effect of exercise on RDQ in LBP patients.

**FIGURE 13:**
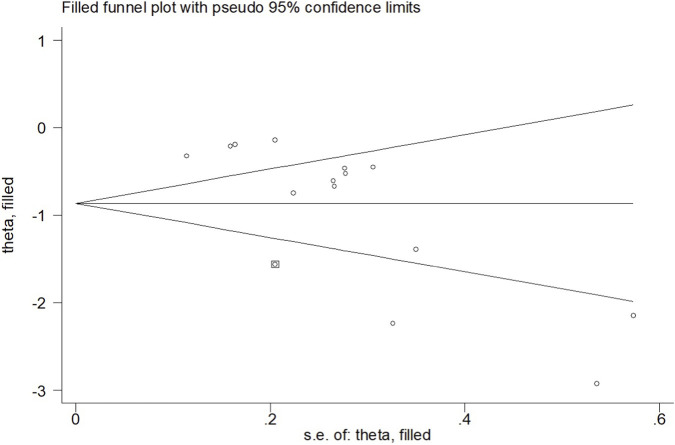
Trim and fill (RDQ).

**FIGURE 14:**
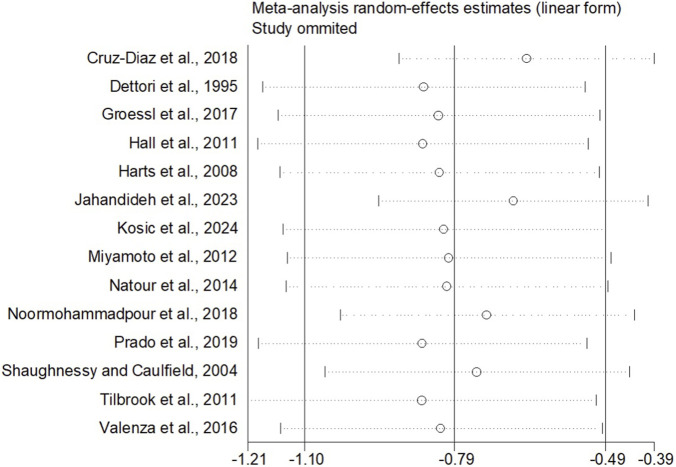
Sensitivity analyses (RDQ).

When disability was assessed with the RDQ, 14 trials enrolling 1,283 participants were included. Four trials achieved high adherence to ACSM recommendations; ten exhibited low or indeterminate adherence. The pooled SMD was −0.77 (95% CI: −1.07, −0.47), indicating a statistically significant improvement in disability. In the high-adherence stratum the SMD was −1.39 (95% CI: −2.51, −0.26), whereas in the low/indeterminate-adherence stratum it was −0.54 (95% CI: −0.76, −0.31). The between-group difference was statistically significant (p < 0.05) (Figure 11). The funnel plot appeared approximately symmetric (Figure 12); however, both Begg’s (P = 0.006) and Egger’s (P = 0.001) tests suggested publication bias. Trim-and-fill imputation added one hypothetical study (Figure 13) and produced a symmetric plot. Sensitivity analysis confirmed that no single study materially influenced the pooled estimate (Figure 14).

## 4 Discussion

This systematic review and meta-analysis examined whether high adherence to ACSM exercise guidelines improves pain and disability in patients with non-specific LBP compared to low or uncertain adherence to these guidelines. Thirty-six trials (2,284 participants) were included.

### 4.1 Positive effects of exercise interventions on LBP

The meta-analysis revealed that exercise interventions with high adherence to ACSM guidelines significantly improve pain and functional disability in individuals with non-specific LBP. This finding aligns with common clinical understanding and previous research (Hayden J. A. et al., 2021; Zhao et al., 2025), confirming the efficacy of structured, guideline-concordant exercise as a non-pharmacological treatment for LBP. A total of 36 RCTs meeting the inclusion criteria were included, encompassing 2,284 patients with nonspecific LBP. The sample population spanned multiple countries, including China, Spain, Iran, the United Kingdom, Brazil, the United States, Portugal, Croatia, and South Korea, indicating the potential generalizability of the findings across diverse geographic and healthcare settings. Although most studies did not systematically report participants' racial or ethnic composition - potentially introducing some population heterogeneity - the overall sample demonstrated broad representativeness.

Consistent evidence indicates that regular, structured exercise interventions yield clinically significant benefits in reducing pain intensity, improving physical function, and enhancing overall quality of life in patients with LBP (Hayden S. J. et al., 2021). The ACSM guidelines provide a standardized framework for exercise prescription encompassing frequency, intensity, time, and type (FITT principles), ensuring that interventions are systematically designed and reproducible. The results demonstrated that adherence to these ACSM guidelines - rather than simply the act of exercising itself - significantly influenced treatment outcomes. Studies with high guideline adherence (defined as ≥75% conformity to ACSM recommendations for exercise dosage) exhibited greater improvements across multiple core outcome measures compared to those with low or uncertain guideline adherence.

In pain assessment, VAS measurements revealed an SMD of −1.98 (95% CI: −2.58, −1.38) in the high-compliance group, indicating a clinically substantial reduction in pain, compared with an SMD of −0.72 (95% CI: −1.01, −0.43) in the low/uncertain-compliance group, which reflects a more modest improvement. This finding is consistent with the conclusions of Geneen et al., who reported a dose-response relationship between structured physical activity and therapeutic efficacy (Geneen et al., 2017). Importantly, our findings suggest that the specific structure and dosage of exercise, as defined by ACSM guidelines, are critical determinants of treatment effectiveness rather than exercise participation alone.

In functional disability assessment, changes in the ODI further underscore the importance of guideline adherence. The high-adherence group demonstrated an SMD of −1.31 (95% CI: −1.81, −0.80) for ODI improvement, significantly outperforming the low-adherence group, which showed an SMD of −0.99 (95% CI: −1.71, −0.28). Similarly, changes in RDQ scores exhibited a comparable trend, with an SMD of −1.39 (95% CI: −2.51, −0.26) in the high-adherence group versus −0.54 (95% CI: −0.76, −0.31) in the low-adherence group, indicating superior recovery in activities of daily living among individuals with higher adherence. These results align with the meta-analysis by Searle et al., which emphasized exercise structure and continuity as key moderators of functional improvement (Searle et al., 2015).

Taken together, exercise therapy demonstrates clear efficacy in the management of LBP, with adherence to ACSM guidelines acting as a critical characteristic that distinguishes more effective from less effective interventions. This underscores the importance of prescribing exercise according to standardized, evidence-based guidelines in clinical practice to achieve optimal intervention effects.

### 4.2 Physiological rationale for ACSM guideline-based exercise in patients with LBP

The superior outcomes observed with high adherence to ACSM guidelines can be understood through multiple physiological and biomechanical mechanisms. Evidence suggests that regular physical activity prescribed at appropriate dosages enhances core muscle strength, improves lumbar joint mobility, and promotes local blood circulation, collectively contributing to pain reduction and improved functional capacity (Gordon and Bloxham, 2016). For instance, aerobic exercises such as brisk walking not only improve cardiorespiratory fitness but also provide better dynamic stabilization of the lumbar spine through enhanced systemic endurance (Kuzu et al., 2025). Engaging in moderate-intensity aerobic exercise three times per week for 30 min per session has been shown to significantly reduce pain intensity in patients with chronic LBP (Meng and Yue, 2015).

Resistance training, particularly targeting the transversus abdominis, multifidus, and gluteal muscle groups, effectively strengthens lumbar segmental stability, offloading stress from the intervertebral discs and facet joints, thereby alleviating pain (Kuzu et al., 2025). A randomized controlled trial demonstrated that patients undergoing an 8-week resistance training program exhibited a mean improvement of 35% in the ODI (Williams and Johnson, 2023).

Furthermore, balance training enhances proprioception and postural control by activating deep stabilizing musculature, which helps reduce compensatory lumbar injuries caused by postural instability (Hlaing et al., 2021). Exercises such as single-leg stance and yoga-based movements have been widely incorporated into LBP rehabilitation programs and have demonstrated favorable clinical outcomes (Yildirim and Gultekin, 2022). Collectively, various forms of exercise exert synergistic effects through distinct physiological mechanisms, not only reducing pain but also improving overall function and quality of life.

### 4.3 Impact of adherence to ACSM guidelines on exercise interventions

The superior outcomes observed in the high-adherence group relative to those with low or uncertain adherence may be attributed to the systematic and standardized nature of the ACSM guidelines. The ACSM-recommended exercise regimens encompass multiple modalities, including aerobic training, flexibility exercises, and resistance training, and provide evidence-based recommendations on frequency, intensity, time, and type of exercise (FITT principles) (Garber et al., 2011). High adherence implies that studies more rigorously implemented these evidence-based dosage parameters, leading to more pronounced improvements in both physiological and functional outcomes.

Studies indicate that moderate-intensity aerobic exercise in accordance with ACSM recommendations - such as 150 min per week of brisk walking - can significantly improve cardiorespiratory fitness and reduce levels of chronic inflammatory markers, thereby alleviating symptoms of LBP (Yildirim and Gultekin, 2022). Additionally, resistance training enhances the strength and stability of the core musculature, which helps reduce mechanical loading on the lumbar spine (Searle et al., 2015).

Flexibility training performed with proper duration (10–30 s per stretch) contributes by improving muscle extensibility and joint range of motion, thereby reducing the incidence of muscle spasms. Taken together, high adherence to ACSM guidelines enables the full realization of benefits from multimodal, structured exercise interventions, leading to superior outcomes in pain relief and functional recovery. Therefore, based on current evidence, high conformity with ACSM guidelines exerts a positive effect on the management of LBP, although findings should be interpreted with caution.

### 4.4 Clinical value of ACSM-Based exercise

Exercise programs based on recommendations from the ACSM hold significant clinical value in the management of LBP. As a safe and effective non-pharmacological intervention, the ACSM guidelines emphasize standardized, individualized, progressive regimens incorporating aerobic exercise, resistance training, and flexibility exercises at specific, evidence-based dosages. These components collectively improve core muscle function and enhance spinal stability, effectively reducing pain and preventing recurrence. Substantial clinical evidence confirms that prescribing exercise according to these structured protocols yields meaningful improvements in patient-reported outcomes and physical function.

Regular implementation of ACSM guideline-concordant exercise prescriptions can significantly reduce pain intensity in patients with LBP, improve physical function and psychological wellbeing, and enhance overall quality of life. Compared with long-term reliance on analgesics, muscle relaxants, or surgical interventions, standardized exercise-based approaches avoid potential adverse effects such as drug dependence, hepatic and renal burden, and surgical risks, offering a safer and more sustainable therapeutic option. Moreover, by enhancing physical function and self-management capacity, ACSM guideline-based exercise therapy helps reduce absenteeism, work disability, and long-term functional limitations associated with LBP, thus alleviating the economic burden on both individuals and society.

Against the backdrop of strained healthcare resources and increasing pressure in chronic disease management, widespread adoption of standardized ACSM exercise protocols not only optimizes treatment pathways for LBP but also significantly reduces healthcare expenditures related to outpatient visits, imaging examinations, and hospitalizations, demonstrating broad societal benefits and long-term public health implications.

### 4.5 Future directions and research limitations

In clinical practice, greater emphasis should be placed on the dissemination and application of standardized guidelines from the ACSM, integrating them into standard management protocols for LBP. Healthcare providers, including physicians and rehabilitation therapists, should develop evidence-based, individualized exercise intervention programs tailored to patients’ specific characteristics - such as age, physical condition, pain severity, degree of functional limitation, and comorbidities - by applying the ACSM-recommended Frequency, Intensity, Time, and Type (FITT) principles. This ensures safe, effective improvements in function and pain reduction.

Furthermore, enhanced multidisciplinary collaboration integrating physical therapy, psychological support, and patient education is essential to maximize rehabilitation outcomes. Future research should further investigate the optimal combination and implementation strategies of ACSM-recommended aerobic exercise, resistance training, and flexibility exercises across different LBP subtypes, including nonspecific LBP, discogenic pain, and spinal degenerative conditions. Critically, future meta-analyses should examine both exercise type (resistance, aerobic, flexibility) and exercise dose to determine potential interactions and optimize clinical utility (Zang et al., 2022; Zang et al., 2026). In addition, attention should be directed toward the application of digital health technologies, such as wearable devices and mobile applications, for remote monitoring of physical activity and the provision of real-time feedback, thereby enhancing patients' self-management capabilities.

Improving prescription of guideline-adherent exercise is crucial to the success of exercise therapy. Future research should further explore the barriers and facilitators to implementing ACSM guidelines in clinical settings and develop targeted behavioral intervention strategies - such as motivational interviewing, goal setting, and social support systems - to promote widespread adoption of standardized exercise protocols. Through the optimization of clinical practice and the advancement of evidence-based research, the standardized and precise implementation of ACSM guidelines in LBP rehabilitation can be promoted, ultimately leading to comprehensive improvements in patients’ quality of life.

Although the findings and contributions of this study are valuable, several limitations should be acknowledged. First, the inclusion criteria across the selected studies were not entirely consistent, with variations in the frequency, intensity, and duration of exercise interventions, which may affect the accuracy and comparability of the results. Second, potential confounding factors exist; for instance, some studies did not adequately control for other variables that might influence pain and functional outcomes, such as patients' psychological status and lifestyle behaviors. Third, although 36 articles were included in this analysis, the number remains relatively limited compared to the vast body of literature on LBP, potentially limiting the generalizability of the findings. Fourth, considerable inter-individual variability exists, as patients may respond differently to exercise interventions, which could also affect the overall applicability of the results.

Fifth, and particularly important, the ACSM-based scoring was performed by authors involved in the study (TT and BZ), which may introduce potential bias despite our efforts to ensure objectivity. Although we employed dual independent assessment with consensus resolution involving a third author (HW) when disagreements occurred, the subjective nature of evaluating guideline adherence cannot be entirely eliminated. Different reviewers might apply scoring criteria with varying degrees of stringency, and the published exercise descriptions in primary studies were often incomplete or ambiguous, requiring interpretive judgment. This subjectivity in scoring may have influenced the classification of studies into high versus low adherence categories, potentially affecting our subgroup comparisons. Future reviews could mitigate this limitation by employing blinded assessment by independent reviewers not involved in other aspects of the review, or by developing more objective, automated methods for evaluating guideline adherence based on standardized reporting templates.

Sixth, we were unable to analyze the effects of specific exercise modes (resistance vs. aerobic vs. flexibility) or components (core strengthening vs. gluteal strengthening vs. stretching) due to inadequate reporting in primary studies. Many studies employed multimodal interventions without clearly delineating the relative contribution of each component, and exercise descriptions were frequently too vague to permit reliable categorization. This limitation prevents us from determining whether certain exercise types are more effective than others when delivered at equivalent ACSM-adherent dosages, or whether specific components interact with dosage to influence outcomes. Future primary studies should employ standardized reporting of exercise parameters according to the FITT framework to enable such analyses.

## 5 Conclusion

Our meta-analysis indicates that exercise prescribed according to ACSM guidelines can decrease pain and function in patients with LBP. Furthermore, strict adherence to the ACSM recommendations (≥75% adherence) was associated with significant decreases in VAS, ODI, and RDQ scores compared to low or uncertain adherence, providing theoretical support for exploring optimal exercise dosage in patients with LBP. These findings demonstrate that the specific structure and dosage of exercise interventions matter - not all exercise is equally effective. However, as some studies did not provide detailed exercise intervention protocols, this conclusion requires further validation through longer-term follow-up and higher-quality RCTs.

## Data Availability

The original contributions presented in the study are included in the article/Supplementary Material, further inquiries can be directed to the corresponding authors.
