# Supplementary material for: Effects of exercise dose based on the ACSM recommendations on pain and disability in non-specific low back pain patients: a systematic review and meta-analysis of randomized controlled trials
Source: Front Physiol. 2026 Mar 11;17:1725132. doi: 10.3389/fphys.2026.1725132 (PMC13013067; doi:10.3389/fphys.2026.1725132)
Supplement: Supplementary file 1 [file Table1.docx]

Supplementary Material

# Supplementary Figures and Tables

**Supplementary TABLE 1.** Search strategy on Web of since.

| #1 | (((((((((((((((((((ALL=(Low back pain)) OR ALL=(Back Pain, Low)) OR ALL=(Back Pains, Low)) OR ALL=(Low Back Pains)) OR ALL=(Pain, Low Back)) OR ALL=(Pains, Low Back)) OR ALL=(Low Back Ache)) OR ALL=(Ache, Low Back)) OR ALL=(Aches, Low Back)) OR ALL=(Back Ache, Low)) OR ALL=(Back Aches, Low)) OR ALL=(Low Back Aches)) OR ALL=(Low Backache)) OR ALL=(Backache, Low)) OR ALL=(Backaches, Low)) OR ALL=(Low Backaches)) OR ALL=(Lower Back Pain)) OR ALL=(Back Pain, Lower)) OR ALL=(Back Pains, Lower)) OR ALL=(Lower Back Pains) |
| --- | --- |
| #2 | (((((((((ALL=(Pain, Lower Back)) OR ALL=(Pains, Lower Back)) OR ALL=(Lumbago)) OR ALL=(Low Back Pain, Mechanical)) OR ALL=(Mechanical Low Back Pain)) OR ALL=(Low Back Pain, Posterior Compartment)) OR ALL=(Low Back Pain, Postural)) OR ALL=(Postural Low Back Pain)) OR ALL=(Low Back Pain, Recurrent)) OR ALL=(Recurrent Low Back Pain) |
| #3 | ((((((((((((((((((ALL=(Exercise)) or ALL=(Exercises)) OR ALL=(Sports)) OR ALL=(Physical Activity)) OR ALL=(Motor Activity)) OR ALL=(Training)) OR ALL=(Endurance training)) OR ALL=(Tai Chi)) OR ALL=(yoga)) OR ALL=(Balance)) OR ALL=(Resistance)) OR ALL=(Flexibility)) OR ALL=(Aerobic)) OR ALL=(Physical exercise)) OR ALL=(Trainings)) OR ALL=(Wuqinxi)) OR ALL=(Baduanjin)) OR ALL=(Yijinjing)) OR ALL=(Pilates) |
| #4 | ((((ALL=(Randomized controlled trial )) OR ALL=(controlled clinical trial)) OR ALL=(randomized)) OR ALL=(placebo)) OR ALL=(randomly) |

**Supplementary TABLE 2.** Search strategy on PubMed.

| #1 | Low back pain[MeSH Terms] |
| --- | --- |
| #2 | (((((((((((((((((((((((((((((Low back pain[Title/Abstract]) OR (Back Pain, Low[Title/Abstract])) OR (Back Pains, Low[Title/Abstract])) OR (Low Back Pains[Title/Abstract])) OR (Pain, Low Back[Title/Abstract])) OR (Pains, Low Back[Title/Abstract])) OR (Low Back Ache[Title/Abstract])) OR (Ache, Low Back[Title/Abstract])) OR (Aches, Low Back[Title/Abstract])) OR (Back Ache, Low[Title/Abstract])) OR (Back Aches, Low[Title/Abstract])) OR (Low Back Aches[Title/Abstract])) OR (Low Backache[Title/Abstract])) OR (Backache, Low[Title/Abstract])) OR (Backaches, Low[Title/Abstract])) OR (Low Backaches[Title/Abstract])) OR (Lower Back Pain[Title/Abstract])) OR (Back Pain, Lower[Title/Abstract])) OR (Back Pains, Lower[Title/Abstract])) OR (Lower Back Pains[Title/Abstract])) OR (Pain, Lower Back[Title/Abstract])) OR (Pains, Lower Back[Title/Abstract])) OR (Lumbago[Title/Abstract])) OR (Low Back Pain, Mechanical[Title/Abstract])) OR (Mechanical Low Back Pain[Title/Abstract])) OR (Low Back Pain, Posterior Compartment[Title/Abstract])) OR (Low Back Pain, Postural[Title/Abstract])) OR (Postural Low Back Pain[Title/Abstract])) OR (Low Back Pain, Recurrent[Title/Abstract])) OR (Recurrent Low Back Pain[Title/Abstract]) |
| #3 | (#1) OR (#2) |
| #4 | Exercise[MeSH Terms] |
| #5 | ((((((((((((((((((Exercise[Title/Abstract]) OR (Exercises[Title/Abstract])) OR (Sports[Title/Abstract])) OR (Physical Activity[Title/Abstract])) OR (Motor Activity[Title/Abstract])) OR (Training[Title/Abstract])) OR (Endurance training[Title/Abstract])) OR (Tai Chi[Title/Abstract])) OR (yoga[Title/Abstract])) OR (Balance[Title/Abstract])) OR (Resistance[Title/Abstract])) OR (Flexibility[Title/Abstract])) OR (Aerobic[Title/Abstract])) OR (Physical exercise[Title/Abstract])) OR (Trainings[Title/Abstract])) OR (Wuqinxi[Title/Abstract])) OR (Baduanjin[Title/Abstract])) OR (Yijinjing[Title/Abstract])) OR (Pilates[Title/Abstract]) "Exercise"[Title/Abstract] OR "Exercises"[Title/Abstract] OR "Sports"[Title/Abstract] OR "physical activity"[Title/Abstract] OR "motor activity"[Title/Abstract] OR "Training"[Title/Abstract] OR "endurance training"[Title/Abstract] OR "tai chi"[Title/Abstract] OR "yoga"[Title/Abstract] OR "Balance"[Title/Abstract] OR "Resistance"[Title/Abstract] OR "Flexibility"[Title/Abstract] OR "Aerobic"[Title/Abstract] OR "physical exercise"[Title/Abstract] OR "Trainings"[Title/Abstract] OR "Wuqinxi"[Title/Abstract] OR "Baduanjin"[Title/Abstract] OR "Yijinjing"[Title/Abstract] OR "Pilates"[Title/Abstract] |
| #6 | (#4) OR (#5) |
| #7 | ((((Randomized controlled trial[Title/Abstract]) OR (controlled clinical trial[Title/Abstract])) OR (randomized[Title/Abstract])) OR (placebo[Title/Abstract])) OR (randomly[Title/Abstract]) |
| #8 | (((#3)) AND (#6)) AND (#7) |

**Supplementary TABLE 3.** Search strategy on Embase.

| #1 | 'low back pain':ab,ti OR 'back pain, low':ab,ti OR 'back pains, low':ab,ti OR 'low back pains':ab,ti OR 'pain, low back':ab,ti OR 'pains, low back':ab,ti OR 'low back ache':ab,ti OR 'ache, low back':ab,ti OR 'aches, low back':ab,ti OR 'back ache, low':ab,ti OR 'back aches, low':ab,ti OR 'low back aches':ab,ti OR 'low backache':ab,ti OR 'backache, low':ab,ti OR 'backaches, low':ab,ti OR 'low backaches':ab,ti OR 'lower back pain':ab,ti OR 'back pain, lower':ab,ti OR 'back pains, lower':ab,ti OR 'lower back pains':ab,ti OR 'pain, lower back':ab,ti OR 'pains, lower back':ab,ti OR lumbago:ab,ti OR 'low back pain, mechanical':ab,ti OR 'mechanical low back pain':ab,ti OR 'low back pain, posterior compartment':ab,ti OR 'low back pain, postural':ab,ti OR 'postural low back pain':ab,ti OR 'low back pain, recurrent':ab,ti OR 'recurrent low back pain':ab,ti |
| --- | --- |
| #2 | exercise:ab,ti OR exercises:ab,ti OR sports:ab,ti OR 'physical activity':ab,ti OR 'motor activity':ab,ti OR training:ab,ti OR 'endurance training':ab,ti OR 'tai chi':ab,ti OR yoga:ab,ti OR balance:ab,ti OR resistance:ab,ti OR flexibility:ab,ti OR aerobic:ab,ti OR 'physical exercise':ab,ti OR 'low backaches':ab,ti OR trainings:ab,ti OR wuqinxi:ab,ti OR baduanjin:ab,ti OR yijinjing:ab,ti OR pilates:ab,ti |
| #3 | 'randomized controlled trial':ab,ti OR 'controlled clinical trial':ab,ti OR randomized:ab,ti OR placebo:ab,ti OR randomly:ab,ti |
| #4 | #1 AND #2 AND #3 |

**Supplementary TABLE 4.** Search strategy on Cochrane.

| #1 | (Low back pain):ti,ab,kw OR (Back Pain, Low):ti,ab,kw OR (Back Pains, Low):ti,ab,kw OR (Low Back Pains):ti,ab,kw OR (Pain, Low Back):ti,ab,kw |
| --- | --- |
| #2 | (Low back pain):ti,ab,kw OR (Back Pain, Low):ti,ab,kw OR (Back Pains, Low):ti,ab,kw OR (Low Back Pains):ti,ab,kw OR (Pain, Low Back):ti,ab,kw |
| #3 | (Back Aches, Low):ti,ab,kw OR (Low Back Aches):ti,ab,kw OR (Low Backache):ti,ab,kw OR (Backache, Low):ti,ab,kw OR (Backaches, Low):ti,ab,kw |
| #4 | (Low Backaches):ti,ab,kw OR (Lower Back Pain):ti,ab,kw OR (Back Pain, Lower):ti,ab,kw OR (Back Pains, Lower):ti,ab,kw OR (Lower Back Pains):ti,ab,kw |
| #5 | (Pain, Lower Back):ti,ab,kw OR (Pains, Lower Back):ti,ab,kw OR (Lumbago):ti,ab,kw OR (Low Back Pain, Mechanical):ti,ab,kw OR (Mechanical Low Back Pain):ti,ab,kw |
| #6 | (Pain, Lower Back):ti,ab,kw OR (Pains, Lower Back):ti,ab,kw OR (Lumbago):ti,ab,kw OR (Low Back Pain, Mechanical):ti,ab,kw OR (Mechanical Low Back Pain):ti,ab,kw |
| #7 | #1 or #2 or #3 or #4 or #5 or #6 |
| #8 | (Exercise):ti,ab,kw OR (Exercises):ti,ab,kw OR (Sports):ti,ab,kw OR (Physical Activity):ti,ab,kw OR (Motor Activity):ti,ab,kw |
| #9 | (Training):ti,ab,kw OR (Endurance training):ti,ab,kw OR (Low Backache):ti,ab,kw OR (yoga):ti,ab,kw OR (Balance):ti,ab,kw |
| #10 | (Resistance):ti,ab,kw OR (Flexibility):ti,ab,kw OR (Aerobic):ti,ab,kw OR (Physical exercise):ti,ab,kw OR (Trainings):ti,ab,kw |
| #11 | (Wuqinxi):ti,ab,kw OR (Baduanjin):ti,ab,kw OR (Yijinjing):ti,ab,kw OR (Pilates):ti,ab,kw |
| #12 | #8 or #9 or #10 or #11 |
| #13 | (Randomized controlled trial):ti,ab,kw OR (controlled clinical trial):ti,ab,kw OR (randomized):ti,ab,kw OR (placebo):ti,ab,kw OR (randomly):ti,ab,kw |
| #14 | #7 and #12 and #13 |
